# Functional outcomes and clinical strength assessment after infraspinatus-sparing surgical approach to scapular fracture: Does it really make a difference?

**DOI:** 10.1186/s10195-018-0509-8

**Published:** 2018-09-05

**Authors:** Giuseppe Porcellini, Paolo Palladini, Stefano Congia, Alessandro Palmas, Giovanni Merolla, Antonio Capone

**Affiliations:** 1 Policlinico Universitario di Modena, Università degli Studi di Modena e Reggio Emilia, Modena, Italy; 2 Centro di chirurgia della spalla e del gomito, Ospedale Civile Cervesi, Cattolica, Italy; 3 Clinica Ortopedica, Ospedale Marino, Università degli Studi di Cagliari, Cagliari, Italy

**Keywords:** Shoulder, Scapular fracture, Judet, Modified Judet, Approach

## Abstract

**Background:**

Surgical treatment of scapular fractures with posterior approach is frequently associated with postoperative infraspinatus hypotrophy and weakness. The aim of this retrospective study is to compare infraspinatus strength and functional outcomes in patients treated with the classic Judet versus modified Judet approach for scapular fracture.

**Patients and methods:**

20 cases with scapular neck and body fracture treated with posterior approach for lateral border plate fixation were reviewed. In 11 of 20 cases, we used the modified Judet approach (MJ group), and in 9 cases we used the classic Judet approach (CJ group). All fractures were classified according to the AO classification system. At follow-up examinations, patients had X-ray assessment with acromiohumeral distance (AHD) measurement, clinical evaluation, active range of motion (ROM) examination, Constant Shoulder Score, and Disability of the Arm, Shoulder and Hand (DASH) Score. Infraspinatus strength assessment was measured using a dynamometer during infraspinatus strength test (IST) and infraspinatus scapular retraction test (ISRT).

**Results:**

Demographic data did not significantly differ between the CJ group and MJ group, except for mean follow-up, which was 4.15 years in the CJ group and 2.33 in the MJ group (*p* < 0.001). All X-ray examinations showed fracture healing. AHD was significantly decreased in the CJ group (*p* = 0.006). We did not find significant differences in active ROM between the MJ and CJ groups in the injured arm (*p* < 0.05). The Constant Score was 75.83 (±14.03) in the CJ group and 82.75 (±10.72) in the MJ group (*p* = 0.31); DASH Score was 10.16 in the CJ group and 6.25 in the MJ group (*p* = 0.49). IST showed mean strength of 8.38 kg (±1.75) in the MJ group and 4.61 kg (±1.98) in the CJ group (*p* = 0.002), ISRT test was 8.7 (±1.64) in the MJ group and 4.95 (±2.1) in the CJ group (*p* = 0.002). Infraspinatus hypotrophy was detected during inspection in six patients (five in the CJ group and one in the MJ group); it was related to infraspinatus strength weakness in IST and ISRT (*p* < 0.001).

**Conclusions:**

Infraspinatus-sparing surgical approach for scapular fracture avoids infraspinatus hypotrophy and external-rotation strength weakness. We suggest use of the modified Judet approach for scapular fracture and to restrict the classic Judet approach to only when the surgeon believes that the fracture is not easily reducible with a narrower exposure.

**Level of evidence:**

Level IV.

## Introduction

Scapula fracture represents a very small part of all fractures [1].

According to Ada et al., fractures located in the glenoid neck and body account for 98 % of all fractures of the scapula, with other less common sites being the acromion, coracoid processes, and scapular spine [2].

They are mostly caused by high-energy trauma and are frequently associated with spine, cranium, and thorax injuries [3].

Development of new techniques has raised considerable interest in operative treatment, even though the vast majority of these fractures are treated conservatively; nowadays, 9.8 % of scapula fractures are treated surgically [4].

Goss described the double lesion of the superior shoulder suspensory complex (SSSC); this condition is defined as lesions of any two structures among the ring composed by the glenoid process, coracoid process, coracoclavicular ligament, distal clavicle, acromioclavicular joint, and acromial process and creates a floating glenohumeral joint that requires operative management [5].

The surgical indication should be primarily related to individual factors: functional demands, ipsilateral injuries, comorbidities, and hand dominance.

Cole et al. [6] categorized, according to available literature, surgical indications based on the degree of deformity and amount of displacement, identifying six operative indications for extraarticular fracture:

1. Medial/lateral displacement >20 mm
2. Angular deformity between the fracture fragments >45°
3. Medial/lateral displacement>15 mm and angulation >30°
4. Glenopolar angle <22° (defined as the angle between the line connecting the uppermost with the lowermost point of the glenoid cavity and the line connecting the uppermost point of the glenoid cavity with the lowermost point of the body of the scapula).
5. Double lesion of the SSSC with displacement of both lesions >10 mm
6. Open fracture

Regarding intraarticular fractures, the indications were: glenohumeral instability, displacement more than 4 mm of articular step-off, or more than 20% of the glenoid involved.

Principles of reduction and fixation such as restoration of articular surface, alignment, and stable internal fixation are well delineated by the Arbeitsgemeinschaft Osteosynthesefragen/Orthopaedic Trauma Association (AO/OTA) [6, 7].

In 1964, Judet described a posterior approach, which required infraspinatus muscle lateral reflection to expose the infraglenoid fossa and the posterior part of the scapular neck; this approach is known as the classic Judet approach [8, 9].

Since then, several approaches have been described with the aim of being more effective and less invasive [10]. In 2004, a modified Judet approach was well described by Obremskey and Lyman [11]. This approach provides a blunt dissection through the internervous interval between the infraspinatus and teres minor, allowing preservation of the infraspinatus in the scapular fossa. It also allows adequate exposure of the posterior glenoid neck and lateral border of the scapula. In addition, treatment of the medial border required localized dissection of the infraspinatus from its origin.

This approach, which preserves the infraspinatus muscle, should not causes weakness of the strength in external rotation and should positively affect functional results, but so far no study has proved or quantified this assumption, comparing results with the classic Judet approach [9, 11].

The aim of this retrospective study is to compare infraspinatus strength and functional outcomes in patients treated in our unit for scapular fracture with classic versus modified Judet approach.

## Materials and methods

### Study population and inclusion criteria

Between January 2010 and December 2016, 117 scapular fractures were surgically treated in our shoulder and elbow department.

**Fig. 1:**
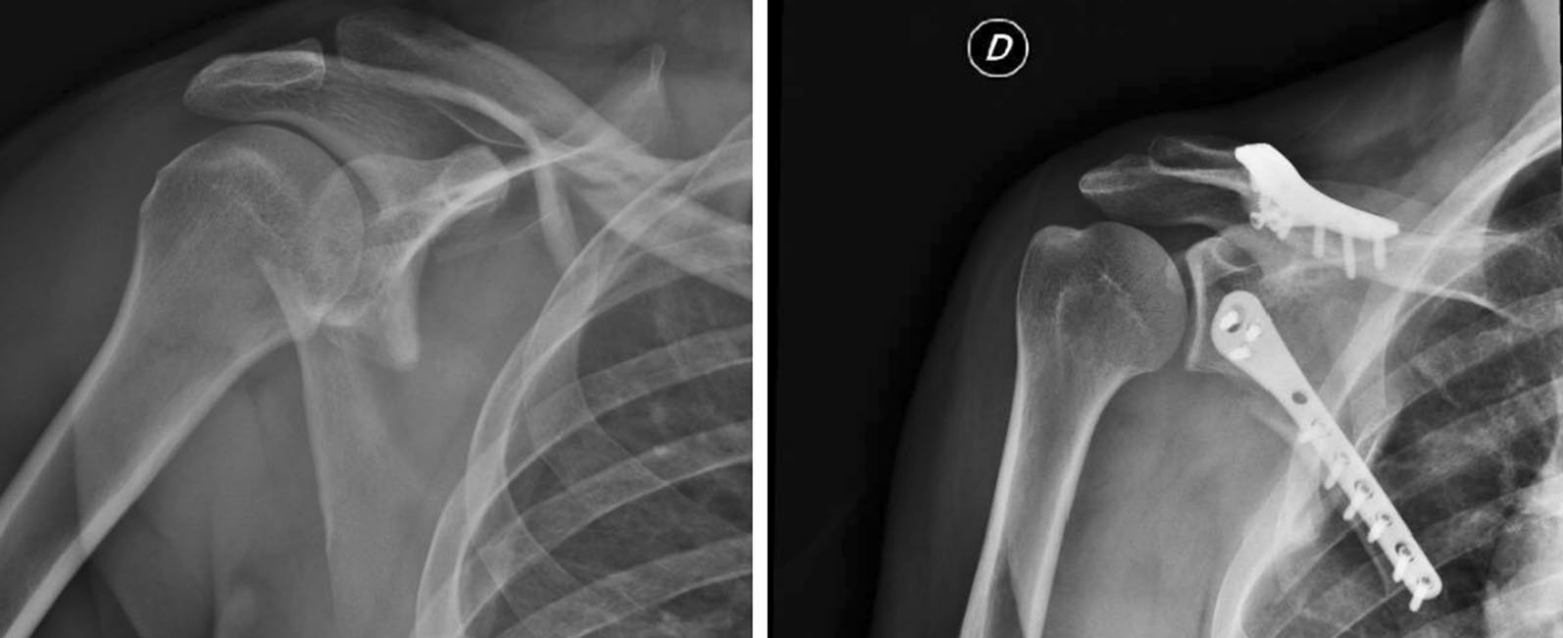
Scapular neck and clavicle fracture (pre- and postoperative X-ray)

**Fig. 2:**
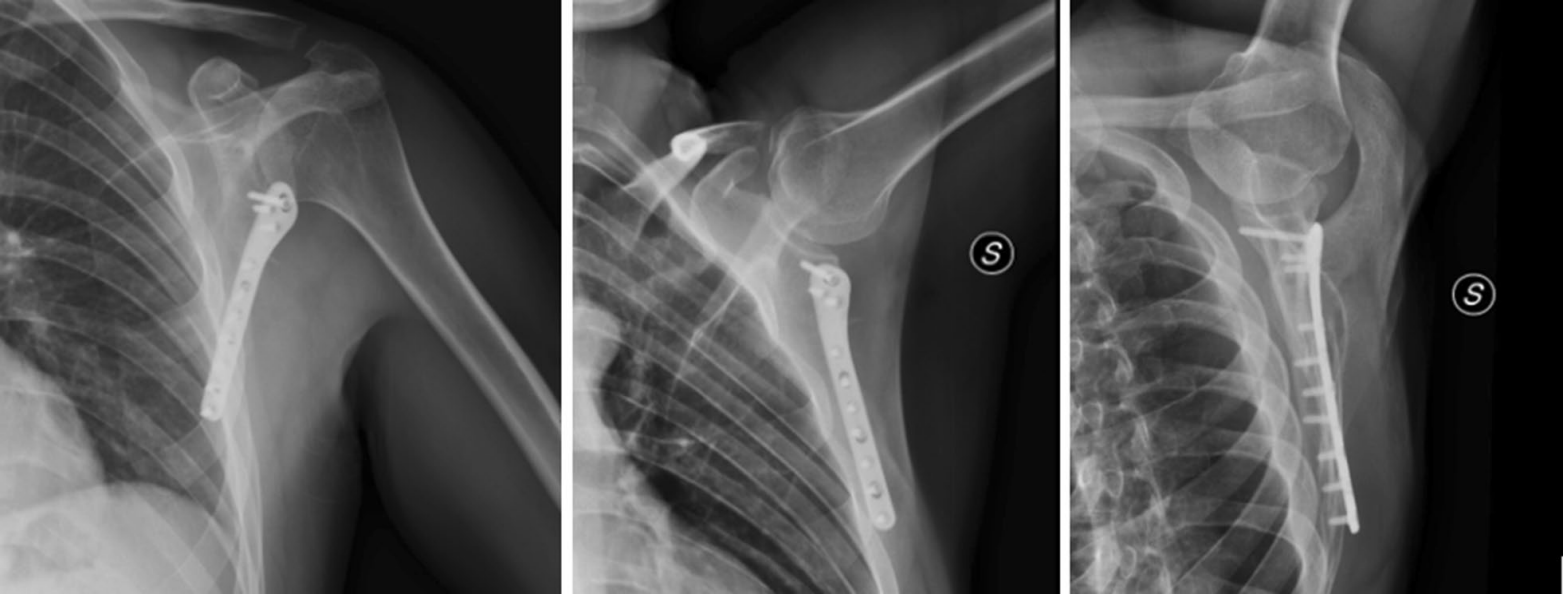
Lateral border plate (X-ray 2 years after operation)

We selected and reviewed 20 patients with scapular neck and body fracture who were treated with posterior approach and lateral plate and screw fixation (Figs. 1 and 2).

The inclusion criteria were:

1. Presence of a scapular extraarticular neck and/or body fracture or intraarticular glenoid fracture extending to the scapular neck
2. Open reduction and internal fixation carried out with posterior approach and lateral plate and screw fixation

Exclusion criteria were:

1. Declared brachial plexus or upper limb nerve palsy in the affected arm preceding or following the trauma
2. Coracoid, acromion, and spine process fractures without scapular neck/body extension
3. Isolated intraarticular glenoid fractures without scapular neck/body extension
4. Use of medial border plate

Five patients did not meet the criteria because they were treated with an L-shaped locking compression medial border plate in addition to the lateral border plate. The medial plate required mandatory localized detachment of the infraspinatus muscle in the superomedial fossa, which could potentially be the cause of infraspinatus weakness, as routinely happens in the classic Judet (CJ) approach.

### Surgical technique and postoperative rehabilitation

All operations were performed by the same senior surgeon (G.P.). In 11 of 20 cases we used the modified Judet approach; in the remaining 9 cases we used the classic Judet approach. All patients were operated under general anesthesia in lateral decubitus position.

In the classic Judet approach, the skin incision was made from the posterolateral corner of the acromion, extending horizontally to the scapular spine and then inferiorly along the medial border. A full-thickness subcutaneous flap was raised off the posterior muscle fascia overlying the deltoid and infraspinatus/teres minor muscle. The deltoid was identified and retracted. Deltoid takedown with partial tenotomy and detachment was executed in both approaches only if better exposure was required. The interval was developed between the posterior deltoid fibers and underlying rotator cuff, then the infraspinatus origin was elevated out of the infraspinatus fossa and reflected laterally towards the spinoglenoid notch. The suprascapular nerve was visualized and secured.

After fracture fixation, the infraspinatus was repositioned in the fossa and repaired with nonabsorbable suture, as was the posterior deltoid to the scapula spine when it was detached.

In the modified Judet approach, we performed an inverted L-shaped skin incision (reverse Judet skin incision), using the same technique as described by Obremskey et al. but with a different skin incision, posterolateral instead of posteromedial, due to the preference of the surgeon (G.P.). The incision started at the scapular spine, extending horizontally and laterally to the posterolateral corner of the acromion, from which the incision became vertically through the lateral border of the scapula. The interval between the infraspinatus and teres minor was identified, and a blunt dissection through this space allowed exposure of the lateral border of the scapula and the posterior glenoid margin. The ascending branch of the circumflex scapular artery was ligated to prevent bleeding.

In case of intraarticular fracture, we executed a capsulotomy perpendicular to the glenoid face, closed with nonabsorbable suture. In case of posterior deltoid detachment, the posterior deltoid was then repaired at the end of the procedure using nonabsorbable suture [11, 12].

All fractures were preoperatively evaluated with trauma series X-ray and tomography scans with three-dimensional reconstructions. All fractures were classified according to the AO classification system by the senior surgeon (G.P.) and one junior orthopedic fellow (S.C.) (Table 1) [13].

**Table 1:** Baseline characteristics: demographic and fracture data

	CJ group	MJ group	*p*-value	Total
No. of patients	6	8	/	14
Follow-up (years)	4.15 (±0.78)	2.33 (±0.72)	< 0.001	3.11 (±1.17)
Age (years)	48.8 (±6.76)	48.5 (±7.5)	0.93	48.6 (±6.92)
Gender	Male = 5, female = 1	Male = 8, female = 0	0.23	Male = 13, female = 1
Weight (kg)	76.1 (±14.14)	74 (±9.3)	0.73	74.9 (±11.17)
Height (cm)	178.5 (±9.02)	173.5 (±8.05)	0.29	175.6 (±8.53)
BMI (kg/m^2^)	23.7 (±2.54)	24.53 (±1.94)	0.5	24.17 (±2.17)
Dominant side	2	4	0.87	/
AO classification	A3 = 1 C1 = 1	A3 = 2 C1 = 1	0.78	/
AO/OTA (14 = scapula)	C2 = 2 C3 = 2	C2 = 3 C3 = 2		
Type of fixation	Acumed lateral border plate = 6	Acumed lateral border plate = 7 Acumed glenoid plate = 1	/	/
Clavicle fracture	4	5	1	9
Rib fractures	4	5	0.87	9
Pneumothorax	2	4	0.53	6

Mean time from trauma to surgery was 5.2 days (range 3–11 days) for the CJ group and 5.5 days (range 3–14 days) for the modified Judet (MJ) group. In 18/20 cases, we used a lateral border anatomically precontoured scapula locking plate with ten holes (Acumed, Acumed LLC, Hillsboro), in one case we used a precontoured glenoid locking plate with four holes (Acumed, Acumed LLC, Hillsboro), and in one case (patient treated in 2010 with CJ approach) we used a contoured Sherman plate with eight holes. In 6/20 cases, we used additional cortical screws out of the lateral plate.

In all cases we used a standard postoperative rehabilitation protocol:

- The arm was immobilized in a sling for 4 weeks;
- Passive mobilization in the scapular plane was allowed from the second postoperative week;
- Active mobilization in a pool was allowed from the fifth postoperative week;
- Dry motions were allowed after 6 weeks;
- Strengthening exercises for scapular muscles and humeral depressors began at the third month in order to balance scapulohumeral rhythm;
- Return to work and sport was allowed between the third and sixth month.

### Study details

The active range of motion of the injured and noninjured arm were measured using a goniometer. Examination of range of motion (ROM) included forward flexion, abduction, and external rotation with the arm at the side and the elbow 90° flexed.

Internal rotation was rated from 0 to 10 based on the ability to raise the hand behind the back (0 = lateral thigh, 10 = T7).

We evaluated infraspinatus fossa filling in order to highlight the presence of muscle hypotrophy.

We carried out Jobe test and scapular retraction test (SRT), as described by Kibler, to assess apparent or real supraspinatus weakness and scapular dyskinesia [14].

Shoulder strength was assessed by Lafayette dynamometer (Lafayette Instruments, Lafayette, IN).

Infraspinatus strength test (IST) [15] and infraspinatus scapular retraction test (ISRT) [16] were performed, as already described, by three measurements in both shoulders.

Range of motion and infraspinatus strength were assessed by two blinded examiners: one junior orthopedic fellow (S.C.) and the senior surgeon (G.P.), in two testing sessions on the patients randomly enrolled with 2 h between sessions. Intra- and interobserver reliability were statistically considered [15, 16].

Constant Score [17] and Disability of the Arm, Shoulder and Hand (DASH) Score [18] were applied to assess clinical outcomes.

All patients underwent X-ray examination (true AP, Y scapular, axillary lateral view); acromiohumeral distance in AP view was calculated as described by Petersson et al. [19].

### Statistical analysis

Descriptive analysis was carried out for both groups.

The CJ and MJ groups were compared for all recorded data using *t* test for independent samples, chi-squared, and Mann–Whitney test, according to the type of variable considered.

Intrapatient comparison of functional outcomes between the injured and uninjured arm was performed with *t*-test for paired samples.

The Cohen kappa (*k*) coefficient was used to measure intra- and interobserver agreement on a nominal scale for IST, ISRT, and active ROM measurements. A value of 0 (*k* = 0) indicates no agreement beyond chance, whereas a value of 1 (*k* = 1) corresponds to perfect agreement.

Stata Intercooled 9.2 software (Stata Corp) was used for all tests. Significance was set at *p* < 0.05. All tests were two-tailed.

## Results

In February 2017, we started examinations on all 20 enrolled patients after institutional board approval. All patients gave informed consent prior to study inclusion. Five patients did not want to participate; one was untraceable. Those who did not agree to participate in the proposed study lived more than 300 km from the hospital; they agreed to undergo a telephonic interview and send us new X-rays and a completed and signed DASH Score [18] form; three of five patients were treated with the MJ approach, and two with the CJ approach.

Eight of 14 patients reviewed at the final follow-up appointment underwent a modified Judet approach (MJ group), while 6 underwent the classic Judet approach (CJ group).

Demographic data, viz. age, gender, BMI, dominant side, and type of work, did not different significantly between the CJ and MJ group, except for mean follow-up, which was 4.15 years in the CJ group and 2.33 years in the MJ group (*p* < 0.001) (Tables 1, 3).

We did not find significant differences between the two groups in terms of type of fracture (AO fracture classification), presence of clavicle fractures, or thorax lesions (Tables 1, 3).

All cases of clavicle fracture were treated with open reduction and internal fixation at the same time as the scapular procedure (Fig. 1).

**Fig. 3:**
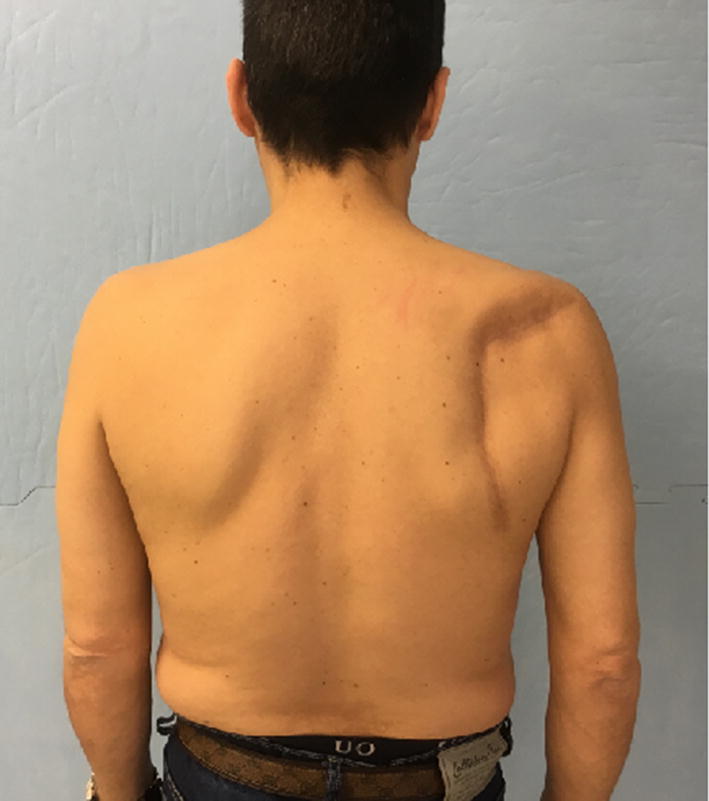
Patient who underwent the classic Judet approach, with evident infraspinatus hypotrophy

**Fig. 4:**
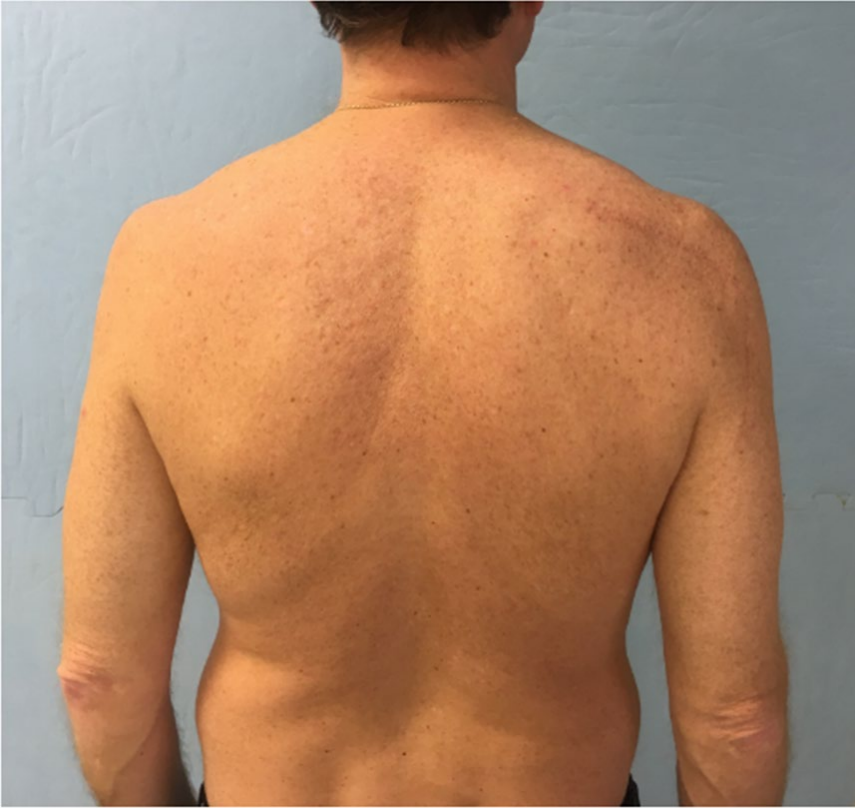
Patient who underwent the modified Judet approach

At follow-up, during inspection of the injured arm, we noticed five patients with infraspinatus hypotrophy in the CJ group and one in the MJ group (*p* < 0.008); we did not find any hypotrophy in the noninjured arm in either group (Figs. 3, 4).

In both groups, we found significant correlation between IST test and infraspinatus hypotrophy (*p* < 0.001) and between ISRT test and infraspinatus hypotrophy (*p* < 0.001).

We did not find any correlation between body mass index (BMI) and infraspinatus hypotrophy (*p* = 0.44).

Infraspinatus strength test in the injured arm showed significant difference between the MJ and CJ groups (Table 2).

**Table 2:** Results

	CJ group	MJ group	*p*-value	Total
Infraspinatus hypotrophy	5	1	0.008	6
IST test noninjured arm (kg)	8.76 (±1.5)	9.22 (±1.18)	0.53	9.02 (±1.29)
IST test injured arm (kg)	4.61 (±1.98)	8.38 (±1.75)	0.002	6.77 (±2.63)
ISRT test noninjured arm (kg)	8.98 (±1.51)	9.28 (±1.09)	0.66	9.15 (±1.24)
ISRT test injured arm (kg)	4.95 (±2.1)	8.7 (±1.64)	0.002	7.09 (±2.61)
AH distance (mm)	7.95 (±1.06)	9.48 (±0.65)	0.006	8.82 (±1.13)
Forward flexion injured arm (°)	146.6 (±36.6)	152.5 (±28.1)	0.74	150 (±32.1)
Abduction injured arm (°)	148.3 (±37.1)	153.7 (±30.6)	0.77	151.4 (±32.3)
External rotation injured arm (°)	61.6 (±18.3)	72.5 (±18.3)	0.29	67.85 (±18.5)
Internal rotation injured arm (points)	7 (±3.03)	7.25 (±2.37)	0.86	7.14 (±2.65)
Forward flexion noninjured arm (°)	176.2 (±6.9)	173.3 (±7.4)	0.69	175 (±7.3)
Abduction noninjured arm (°)	176.2 (±6.9)	171.6 (±8.9)	0.46	174.3 (±8.2)
External rotation noninjured arm (°)	81.2 (±6)	80 (±5.7)	0.61	80.7 (±5.9)
Internal rotation noninjured arm (points)	9 (±1.4)	9.3 (±0.94)	0.74	9.14 (±1.24)
Constant Shoulder Score (points)	75.83	82.75	0.33	79.78
DASH Score (points)	10.16	6.25	0.6	7.92

IST test in the injured arm was 4.61 (±1.98) in the CJ group and 8.38 (±1.75) in the MJ group (*p* < 0.002); ISRT test in the injured arm was 4.95 (±2.1) in the CJ group and 8.7 (±1.64) in the MJ group (*p* < 0.002).

We did not find significant differences in active ROM between the MJ and CJ groups in the injured (Table 2) or noninjured arm.

Clinical outcomes were measured and compared in the CJ and MJ group; Constant Score was 75.83 (±14.03) in the CJ group and 82.75 (±10.72) in the MJ group (*p* = 0.31); DASH Score was 10.16 in the CJ group and 6.25 in the MJ group (*p* = 0.49) (Tables 2, 4).

We did not find complications, except for one superficial wound infection that was treated successfully with intravenous antibiotics and one patient who developed a keloidal scar; no patients underwent reoperation.

We found three cases of real supraspinatus weakness and one case of scapular dyskinesia.

Two of the three patients with supraspinatus weakness reported history of shoulder pain that preceded the trauma; scapular dyskinesia was detected in the patient with shortest follow-up time (12 months).

All patients in both groups, except for two of them who were unemployed for unrelated reasons, returned to work with no severe limitations (seven manual workers and five nonmanual workers). Examining DASH Score forms, we found five patients who reported mild–moderate difficulties at work, four (80%) from the CJ group and one (20%) from the MJ group (Tables 3, 4).

**Table 3:** Patient follow-up data

*N*	Group	Follow-up (years)	Age (years)	Gender	Type of work	Mechanism of injury	BMI (kg/cm^2^)	Dominant arm	AO/OTA classification	Clavicle fracture	Rib fracture	PNX
1	MJ	1.6	44	M	MW	MVC	25	Yes	C2	Yes	Yes	Yes
2	MJ	2.1	35	M	MW	BC	23.5	Yes	C3	No	Yes	No
3	MJ	2.8	59	M	NMW	MVC	21.5	No	C3	No	No	No
4	MJ	2.9	54	M	NMW	MVC	25.1	No	C2	No	Yes	Yes
5	MJ	3.1	46	M	MW	MVC	28.4	Yes	A3	Yes	No	No
6	MJ	1	53	M	NMW	BC	23.7	Yes	C1	No	Yes	Yes
7	MJ	2.8	45	M	MW	MVC	24.2	Yes	C2	No	No	No
8	MJ	2.4	52	M	U	FFH	24.8	No	A3	Yes	Yes	Yes
9	CJ	4.2	58	M	MW	MVC	24.6	Yes	C3	No	Yes	No
10	CJ	2.8	50	M	MW	LHT	24.5	Yes	C2	No	Yes	No
11	CJ	4.3	52	M	NMW	MVC	26.3	Yes	A3	Yes	Yes	Yes
12	CJ	3.8	45	M	MW	MVC	25.9	No	C1	Yes	No	No
13	CJ	4.8	50	F	NMW	MVC	20.2	Yes	C2	No	Yes	Yes
14	CJ	5	38	M	U	BC	24.1	No	A3	Yes	No	No

*N* patient number, *U* unemployed, *MW* manual worker, *NMW* nonmanual worker, *MVC* motor vehicle collision, *BC* bicycle collision, *LHT* low-energy trauma, *FFH* fall from height, *PNX* pneumothorax

**Table 4:** Patient follow-up results

*N*	Group	Constant Score	DASH Score	Return to work	Infrasp. hypotrophy	IST test (kg) IA/UA	ISRT test (kg) IA/UA	AHD (mm)	FF IA/UA	ABD IA/UA	ER IA/UA	IR IA/UA
1	MJ	75	8	Yes	No	8.6/9.1	9.2/8.6	9.6	120/170	110/160	80/80	4/8
2	MJ	92	0	Yes	No	8.7/9.2	9.3/8.7	8.3	170/180	170/170	90/90	10/6
3	MJ	79	7	Yes	No	8.3/8	7.9/8.7	9.7	150/180	160/180	70/80	8/10
4	MJ	97	0	Yes	No	7.2/7.9	7.8/7.9	10.1	180/180	180/180	80/90	10/10
5	MJ	89	3	Yes	No	9.7/10.6	10.6/10.5	9.8	170/160	170/180	80/80	8/10
6	MJ	63	26	Mild–moderate difficulty	Yes	5.1/8.6	5.4/9.1	8.9	100/180	100/180	30/70	4/10
7	MJ	81	4	Yes	No	11.1/11.3	10.1/11.2	9.2	160/180	170/180	70/80	8/10
8	MJ	86	2	NA	No	8.4/9.1	9.3/9.6	10.3	170/180	170/180	80/80	6/8
9	CJ	76	5	Mild–moderate difficulty	Yes	3.4/7.6	3.9/8.6	7.8	130/170	130/160	70/80	6/10
10	CJ	84	4	Mild–moderate difficulty	No	8.3/7.8	8.9/7.3	7.2	170/180	170/180	80/80	10/10
11	CJ	62	18	Mild–moderate difficulty	Yes	5.1/8	5.2/8.3	9.8	160/160	170/180	40/80	6/8
12	CJ	86	2	Yes	Yes	2.6/7.9	2.9/8.3	6.7	160/180	170/170	60/80	8/8
13	CJ	56	32	Mild–moderate difficulty	Yes	4.1/11.2	4.9/11.6	7.9	80/170	80/160	40/90	2/10
14	CJ	91	0	NA	Yes	4.2/10.1	3.9/9.8	8.3	180/180	170/180	80/70	10/10

*NA* not applicable, *IA* injured arm, *UA* uninjured arm, *AHD* acromiohumeral distance, *FF* forward flexion, *ABD* abduction, *ER* external rotation, *IR* internal rotation

Comparing the injured and noninjured arm in both groups, we found that the IST test showed mean strength of 6.77 kg (±2.63) in the injured arm and 9.02 kg (±1.2) in the noninjured arm (*p* = 0.005); ISRT test was 7.09 (±2.61) in the injured arm and 9.1 (±1.24) in the noninjured arm (*p* = 0.015).

We also found significant differences between the injured and noninjured arm in terms of active ROM: mean abduction 151.4° (±32.3°) in injured arm and 174.2° (±8.5°) in noninjured arm (*p* = 0.008); mean forward flexion 150° (±30.88°) in injured arm and 175° (±7.6°) in noninjured arm (*p* = 0.087); external rotation 67.8° (±18.4°) in injured arm and 80.7° (±6.15°) in noninjured arm (*p* = 0.02); internal rotation 7.14 (±2.56) points in injured arm and 9.14 (±1.29) in noninjured arm (*p* = 0.24).

X-ray showed that all fractures healed without malunion and hardware mobilization (Fig. 2).

We found a difference in terms of acromiohumeral distance between the two groups: 7.95 mm (±1.06) in the CJ group and 9.48 mm (±0.65) in the MJ group; only one distance was calculated as less than 7 mm (6.7 mm) (Tables 2, 4).

The interobserver reliability yielded *k* values of 0.82 for ROM assessment and 0.85 for strength measurement; intrarater reliability was 0.79 for ROM assessment and 0.82 for strength measurement.

In the five patients evaluated only with telephonic interview, we did not found major complications (pseudoarthrosis, glenohumeral arthrosis, screw and/or plate mobilization) at the X-ray control; the mean DASH Score according to the forms completed and sent to our clinic was 8.6 points.

## Discussion

Fracture of the scapula has been managed nonoperatively for decades, mainly due to its muscular envelope and mobility on the thoracic cage [20–22].

However, with time, it has been seen by several authors that the functional outcome of patients affected by a displaced fracture following conservative management was not satisfactory; these patients later developed poor and painful shoulder motion [2, 4, 23, 24].

Therefore, an increasing trend towards surgical management of scapular fracture has been described, particularly for those fractures associated with glenoid neck and body fractures and with lesions of the superior shoulder suspensory complex [4, 5].

Following open reduction and internal fixation, displacement, angulation, and glenopolar angle are corrected to achieve good functional outcomes [25–27].

Scapular fractures have complex patterns, but there are predictable satisfactory bone stock areas and landmarks helpful in planning the surgical approach. Landmarks (lateral scapula border, spinoglenoid notch, inferior pole, superomedial corner of infraspinatus fossa, posterolateral acromion, inferior glenoid neck, posterior and inferior glenoid margin) allow safe and optimum exposure without complete denuding the bone; they should be used differently according to the pattern of the fracture. For the internal fixation, an approach to bone with adequate thickness has to be planned; satisfactory bone stock areas are glenoid neck, acromion, scapular spine, and lateral scapular border [28, 29].

Since the majority of scapula fractures involve the scapular body and neck region, the posterior approach is commonly used for internal fixation [2, 30].

Among the posterior approaches, the classic Judet approach has been used for years for exposure and internal fixation of fracture [8].

Due to its extensive muscular dissection and increased postoperative morbidity, several authors have evolved various modifications of the classic Judet approach, including the modified Judet and minimally invasive approaches.

Gauger et al. described a minimally invasive approach capable of exposing the posterior body, neck, and glenoid. They evaluated the results in seven patients, reporting mean DASH Score of 8.1. In that series, muscle strength and motion returned to equivalency to the uninjured arm.

The authors also reviewed 10 different posterior approaches reported in literature from 1984 to 2011 as variants of the classic Judet approach, and reported results and limitations [10].

The MJ approach, as described by Obremskey and Lyman in 2004, is now a widely recommended approach involving the neurovascular interval between the infraspinatus and teres minor. This interval allows a safe surgical dissection plane [31].

This approach allows excellent fracture visualization, hardware placement, and functional return. In addition, it helps surgeons to achieve good and reliable access to bony landmarks necessary for fracture fixation with minimal exposure of the fracture fragments [32].

Harmer et al. showed, in a cadaveric study, that the MJ approach exposes only 20 % of the area visualized with CJ but allows similar access to important landmarks for reduction and fixation, minimizing soft-tissue excision [9].

A study of Salassa et al. pointed out that posterior approach to the scapula without deltoid takedown allowed 91 % of exposure of the bony scapula obtained by removing the deltoid muscle; those author suggested to only proceed with takedown if additional exposure is needed [33].

We preserved the posterior deltoid in the majority of cases presented, in order to minimize soft-tissue damage; we did not find any cases of posterior deltoid hypotrophy, but we cannot say that the deltoid detachment will not affect the results.

The purpose of this study is to prove that a infraspinatus-sparing approach helps recovery with well-maintained muscle strength in the postoperative period. In our study, the age, gender, BMI, and dominant side were not significantly different between the CJ and MJ groups.

We found infraspinatus weakness in patients of the CJ group when compared with the MJ group, which was statistically significant (*p* < 0.05). The main reason for such weakness is the extensive elevation and mobilization of the infraspinatus muscle belly from the fossa to expose the fracture. Other reasons for weakness were also ruled out. The first common reason is entrapment of the suprascapular nerve in the fracture line in cases of scapular neck fracture. In such cases, the nerve was completely mobilized along its course. The second reason is stretching of the nerve intraoperatively during exposure of the fracture. In such cases, care was taken by constant visualization of the nerve during the procedure. Thus, to visualize the nerve completely up to the spinoglenoid notch, mobilization of the infraspinatus muscle is required, which is very well done in the CJ approach. The third reason is inadequate reinsertion of the muscle. The fourth reason, though less common, can be insufficient postoperative rehabilitation. This final reason for weakness was observed in one of our patients treated by the MJ approach. However, this patient had shorter duration of follow-up to comment upon the significance of his hypertrophy.

We did not find significant differences in active ROM between the MJ and CJ groups in the injured arm, but four of five (80 %) of the manual workers who presented mild–moderate difficulties on DASH Score belonged to the CJ group, and this may be due to significant differences in external rotation strength between the two groups.

Novè-Josserand proved that AHD was associated with rotator cuff tear involving the infraspinatus and fatty degeneration of the supraspinatus or infraspinatus [34].

Moreover, Goutallier et al. stated that AHD inferior to 6 mm was seen only in cases of total full-thickness infraspinatus tears associated with severe fatty degeneration [35].

We found a statistical difference in acromiohumeral distance between the CJ and MJ group (*p* = 0.006). In no case was AHD less than 6 mm; this could hypothetically be attributable to infraspinatus weakness, hypertrophy, and degeneration occurring in patients treated with the classic Judet approach. However, no study has proved this to date, and we did not carry out X-ray of the noninjured arm in order to partially reduce the inaccuracy of the method.

We did not perform intramuscular electromyography, as we considered it invasive even if relevant for our purpose. Moreover, we did not perform surface electromyography, as it has been proved that it does not accurately show the activity of the infraspinatus muscle [36, 37].

We acknowledge limitations to our study. Even though it shows statistically significant data, since our sample size is small, the power of statistical tests is very low, hence further studies are required. Secondly, there is a difference between the two groups in terms of mean follow-up; this is due to the progressive increase of the modified Judet approach in our practice.

Scapular fractures are rare events, therefore collecting a large number of cases is challenging even in major shoulder units. Moreover, all cases are hardly comparable because of their unique characteristics.

We started our practice only using the CJ approach for all cases. Over the years, we changed our practice to use both the MJ and CJ approaches, preferring the CJ approach when we thought that complete exposure of the scapula was indispensable. This choice was not made based on any guideline but only on the experience of the surgeon (G.P.).

The results of this study show that patients with scapular fracture treated with infraspinatus-sparing approach presented infraspinatus hypotrophy and weakness less frequently; they also presented a (not statistically significant) tendency for better functional outcomes in terms of DASH Score, Constant Score, and limitations at work.

Further studies are required to understand when use of the classic Judet approach is unavoidable.
